# Lactate is THE target for early resuscitation in sepsis

**DOI:** 10.5935/0103-507X.20170021

**Published:** 2017

**Authors:** Jan Bakker

**Affiliations:** 1 Department of Intensive Care Adults, Erasmus MC University Medical Center - Rotterdam, Netherlands.; 2 Division of Pulmonary, Allergy, and Critical Care Medicine, Columbia University Medical Center - New York, United States.; 3 Division of Pulmonary, Sleep Medicine and Critical Care, New York University - Langone Medical Center - New York, United States.; 4 Department of Intensive Care, Pontificia Universidad Catolica de Chile, Santiago, Chile.

## INTRODUCTION

The resuscitation of patients in sepsis is a challenge for many reasons. One of the important questions is: Who needs what kind of resuscitation? In the present guidelines, resuscitation is mostly directed at patients with a high risk of mortality. In this review, I will discuss the value of using lactate levels to identify patients who might benefit from treatment and how to use sequential lactate levels in the process.

## THE CLINICAL SCENARIO

Although lactate has been advocated as a marker of tissue hypoperfusion by the Surviving Sepsis Campaign guidelines when it rises above 1.0mmol/L,^(1)^ aggressive fluid resuscitation is only recommended for patients with a lactate level above 4.0mmol/L, due to its association with high mortality.^(2)^ However, the origin of hyperlactatemia and its treatment might be more complex. First, it is important to realize that tissue hypoperfusion does not cause a rise in lactate until the decrease in oxygen delivery to the tissues (as an effect of hypoperfusion) reaches a critical point, where it is insufficient to meet the oxygen demand of the tissues, causing cellular dysoxia to occur and lactate levels to increase.^(3)^ Second, as the clearance capacity of the liver almost disappears in sepsis,^(4)^ persistently increased lactate levels may not be related to tissue dysoxia. Finally, other factors in sepsis might contribute to increased lactate levels in the presence of adequate tissue oxygen delivery.^(5)^ Nevertheless, lactate is an important marker of the patient's response to the initiated therapy. In ALL forms of acute circulatory failure, a decrease in lactate levels is associated with a more favorable outcome.^(6)^ Thus, from this we can form our first conclusion: If lactate levels DO NOT decrease following the initiation of treatment, something is wrong.

Although there are many reasons why patients with sepsis might have increased lactate levels, in the early presentation of these patients, inadequate oxygen delivery is the most likely cause. It is also the only cause we can effectively treat when we exclude intoxications, inborn errors of metabolism and other metabolic causes of increased lactate levels.^(5)^ The initial treatment of hyperlactatemia in patients with sepsis should be directed at improving tissue oxygen delivery. This is most effectively accomplished by improving global blood flow, which aims to improve microcirculatory perfusion. Other measures that could be used simultaneously are improving arterial oxygen saturation, improving hemoglobin levels and decreasing oxygen demand. When started immediately upon admission to the ICU, this package will not only effectively decrease lactate levels; it will also improve survival by 20%.^(7)^ Although patients in afore mentioned study^(7)^ had a lactate concentration at or above 3.0mmol/L, it is conceivable that patients with lower concentration levels might also benefit, as lactate levels between 2.0 - 3.9mmol/L in patients with suspected infection are associated with significant mortality, even in the absence of hypotension.^(8)^ The concept of using the treatment package from the study by Jansen et al.^(7)^ only in the early resuscitation period (first 8 hours of ICU admission) was recently confirmed in a study on septic shock survivors. Hernandez et al.^(9)^ assessed the normalization ratio of lactate and showed that a biphasic curve existed. In the early hours (first 6 hours), lactate levels normalized rapidly following the initiation of therapy. In the second phase (up to 24 hours), the normalization was much slower. In the end, 50% of the patients (all survivors) had increased lactate levels at 24 hours after the initiation of treatment. The authors speculated that, early in the course of sepsis, the increased lactate levels quickly responded to improvements in tissue oxygen delivery (the main effect of increasing cardiac output by fluid resuscitation and improving perfusion pressure by using vasopressors). This phase might thus represent a flow-dependent phase of hyperlactatemia whereas, in the later phase, the increased lactate levels are probably more related to other factors. This allows a second conclusion on the use of lactate levels in patients with sepsis: Increased lactate levels should be seen as the consequence of inadequate tissue oxygenation for only a limited time in the early course of sepsis. In addition, driving the patient to normal lactate levels with the continued resuscitation of tissue perfusion/oxygenation might not be effective any longer.

The ultimate goal of resuscitation is to restore microcirculatory perfusion, not macro hemodynamics.^(10)^ However, current guidelines/protocols are still mostly directed towards macrocirculatory parameters, such as blood pressure, using aggressive fluid resuscitation. Although recent trials on the use of early goal directed therapy (EGDT) have shown no benefit over usual care,^(11)^ we should recognize that the resuscitation of sepsis patients has already changed significantly. In the recent EGDT studies, fluid resuscitation was already done in the majority of patients. Even in the lactate study by Jansen et al.,^(7)^ lactate levels had the exact same trajectory in the control group patients compared to the protocol patients, despite the fact that the treatment team was unaware of the actual levels. This brings up a relevant question: If the lactate levels were not different between the two groups, why did the patients in the lactate oriented group have better survival? There were few differences in the variables collected in the study that could explain this effect. On average, the patients in the protocol group were treated with approximately 500mL more fluids in the treatment period and over 1L less fluids in the observation period (8 - 72 hours after initiation of treatment). In addition, the use of nitroglycerin to improve microcirculatory perfusion (as demanded by protocol in the protocol group) was more present in the protocol group than the control group (43% *versus* 20% of the patients, respectively). Although this did not result in differences in lactate levels, this adjustment in therapy might have had a significant effect in the patients who really needed the extra fluids and vasodilators when their lactate levels did not decrease as projected (20% decrease per 2 hours in the protocol group). Second, the use of less fluids in the observation period might have resulted in less morbidity associated with fluid overload.^(12-14)^

One aspect, related to a comment made earlier, was not captured in the study. The goal in the protocol group was to decrease lactate by at least 20% per 2 hours; upon failure to meet this goal, a reassessment of the current treatment was initiated and, in some patients, additional diagnostic procedures (CT-scan, echo, etc.) were initiated and therapy was adjusted (laparotomy, change of antibiotic regimen, etc.).

**Figure 1:**
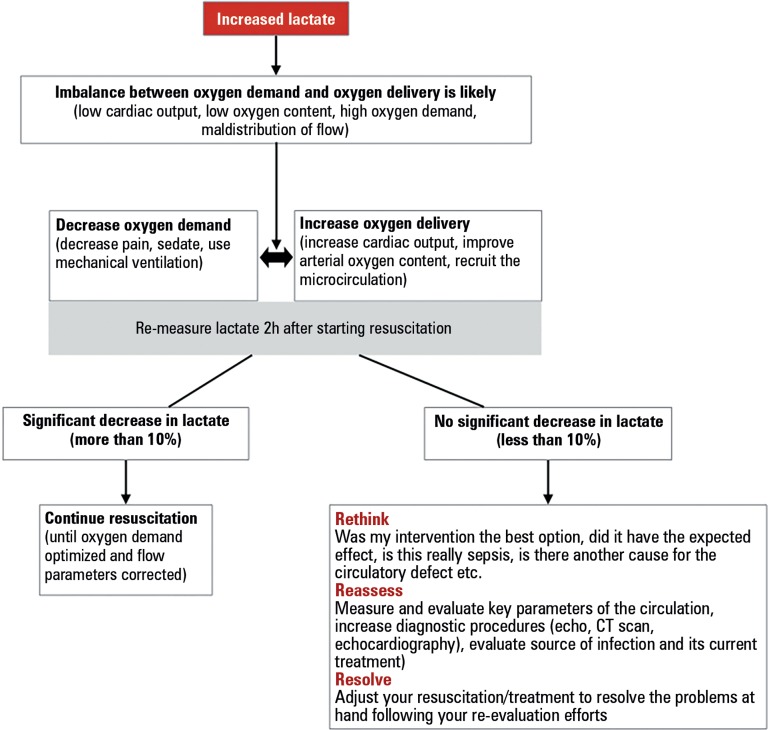
How to use lactate in resuscitating sepsis associated circulatory dysfunction.

Therefore, given the above arguments, we can conclude that, in patients with sepsis, early resuscitation of the circulation aimed to improve the balance between oxygen delivery and oxygen demand, thereby restoring tissue oxygenation using a multimodal approach that is effective in improving survival. Several important factors should be taken into account when using this approach. First, this approach should be used for a limited time (current evidence suggests 6 - 8 hours). Although the original study^(7)^ used a lactate level above 3.0mmol/L, studies suggest that this regimen might be effective in all sepsis patients with increased lactate levels (above 2.0mmol/L). Following the start of resuscitation, lactate levels should decrease rapidly if the balance between oxygen demand and oxygen delivery indeed improves (Figure 1). Therefore, frequent measurements (at least every 2 hours) should be part of the resuscitation protocol. If the therapeutic measures do not result in a rapid decrease in lactate levels, then RETHINK, REASSESS and RESOLVE.
